# Pigmented villonodular synovitis does not influence the outcomes following cruciate-retaining total knee arthroplasty: a case-control study with minimum 5-year follow-up

**DOI:** 10.1186/s13018-020-01933-x

**Published:** 2020-09-07

**Authors:** Wei Lin, Yike Dai, Jinghui Niu, Guangmin Yang, Ming Li, Fei Wang

**Affiliations:** Department of Orthopedic Surgery, Third Hospital of Hebei Medical University, No. 139 Ziqiang Road, Shijiazhuang, 050051 Hebei China

**Keywords:** Pigmented villonodular synovitis, Osteoarthritis, Cruciate-retaining, Total knee arthroplasty

## Abstract

**Background:**

Pigmented villonodular synovitis (PVNS) is a rare synovial disease with benign hyperplasia, which has been successfully treated with total knee arthroplasty (TKA). The purpose of this study was to investigate the middle-term follow-up outcomes of cruciate-retaining (CR) TKA in patients with PVNS.

**Methods:**

From January 2012 to December 2014, a retrospective study was conducted in 17 patients with PVNS who underwent CR TKA as PVNS group. During this period, we also selected 68 patients with osteoarthritis who underwent CR TKA (control group) for comparison. The two groups matched in a 1:4 ratio based on age, sex, body mass index, and follow-up time. The range of motion, Knee Society Score, revision rate, disease recurrence, wound complications, and the survivorship curve of Kaplan-Meier implant were assessed between the two groups.

**Results:**

All patients were followed up at least 5 years. There was no difference in range of motion and Knee Society Score between the two groups before surgery and at last follow-up after surgery (*p* > 0.05). In the PVNS group, no patients with the recurrence of PVNS were found at the last follow-up, one patient underwent revision surgery due to periprosthetic fracture, and three patients had stiffness one year after surgery (17.6% vs 1.5%, *p* = 0.005; ROM 16–81°), but no revision was needed. At 7 years, the implant survivorship was 90.0% in the PVNS group and 96.6% in the control group (*p* = 0.54).

**Conclusions:**

This study demonstrated that the function of patients with PVNS who underwent CR TKA had been significantly improved, and the survival rate of implants in these patients was similar to the patients with OA. Consequently, the patients with PVNS who underwent CR TKA might be an achievable option. However, these patients should pay more attention to the occurrence of postoperative stiffness complications.

## Background

Pigmented villonodular synovitis (PVNS) was initially described by Jaffe in 1941 as a benign, locally aggressive disease characterized by excessive proliferation of histiocytes in the synovium [1, 2]. It is characterized by mechanical symptoms, local recurrence, and knee stiffness which can lead to severe joint destruction [3]. PVNS mainly affects young people aged 30–40, and there is no difference in gender preference [4]. The knee is the most common diseased joint [5, 6]. Prior to joint destruction, arthroscopic or open surgery is the main treatment. However, once the joint destruction occurs, there are few options for pain relief and functional improvement [3, 7, 8].

Total knee arthroplasty (TKA) has been successfully used to improve the function and relieve pain in patients with PVNS. According to some articles, the survival rate of prosthesis ranges from 80 to 85% in patients with PVNS who have been followed up for more than 10 years [9]. In addition, the incidence of postoperative complications of TKA also provided acceptable results in a large number of patients diagnosed with PVNS [10]. However, it is still controversial to retain or substitute posterior cruciate ligament (PCL) in TKA when treated with patients with PVNS. Due to limited case studies of the knee PVNS, there were few data have been available on the clinical outcomes of patients with PVNS who underwent cruciate-retaining (CR) TKA. Moreover, in previous studies, there was a lack of evaluation of the results of control and middle-term study.

Therefore, the purpose of this study was to investigate the disease recurrence rate and the middle-term clinical outcomes of patients with PVNS who underwent CR TKA and compared with a group of patients with knee osteoarthritis (OA) who underwent CR TKA.

## Methods

### Study cohort

With the approval of the Institutional Review Committee, we conducted a retrospective study from January 2012 to December 2014. We included 17 patients who were diagnosed as knee PVNS in the PVNS group. To improve the reliability of this research, we used a 1:4 ratio with regard to age, sex, body mass index (BMI), and follow-up time to select 68 patients with knee OA who underwent CR TKA (Control group) for comparison. In the PVNS group, according to Jaffe’s classification, all patients were diagnosed as diffuse PVNS by pathology [11]. The inclusion criteria were (i) patients with unilateral knee PVNS in the PVNS group, (ii) patients with unilateral knee OA in the control group, (iii) varus or valgus deformity < 20°, and (iv) flexion-contracture deformity < 20°. Patients who had neurological problems, anticoagulant therapy, and revision TKA were excluded.

### Clinical and radiographic features of patients with PVNS before CR TKA

**Fig. 1:**
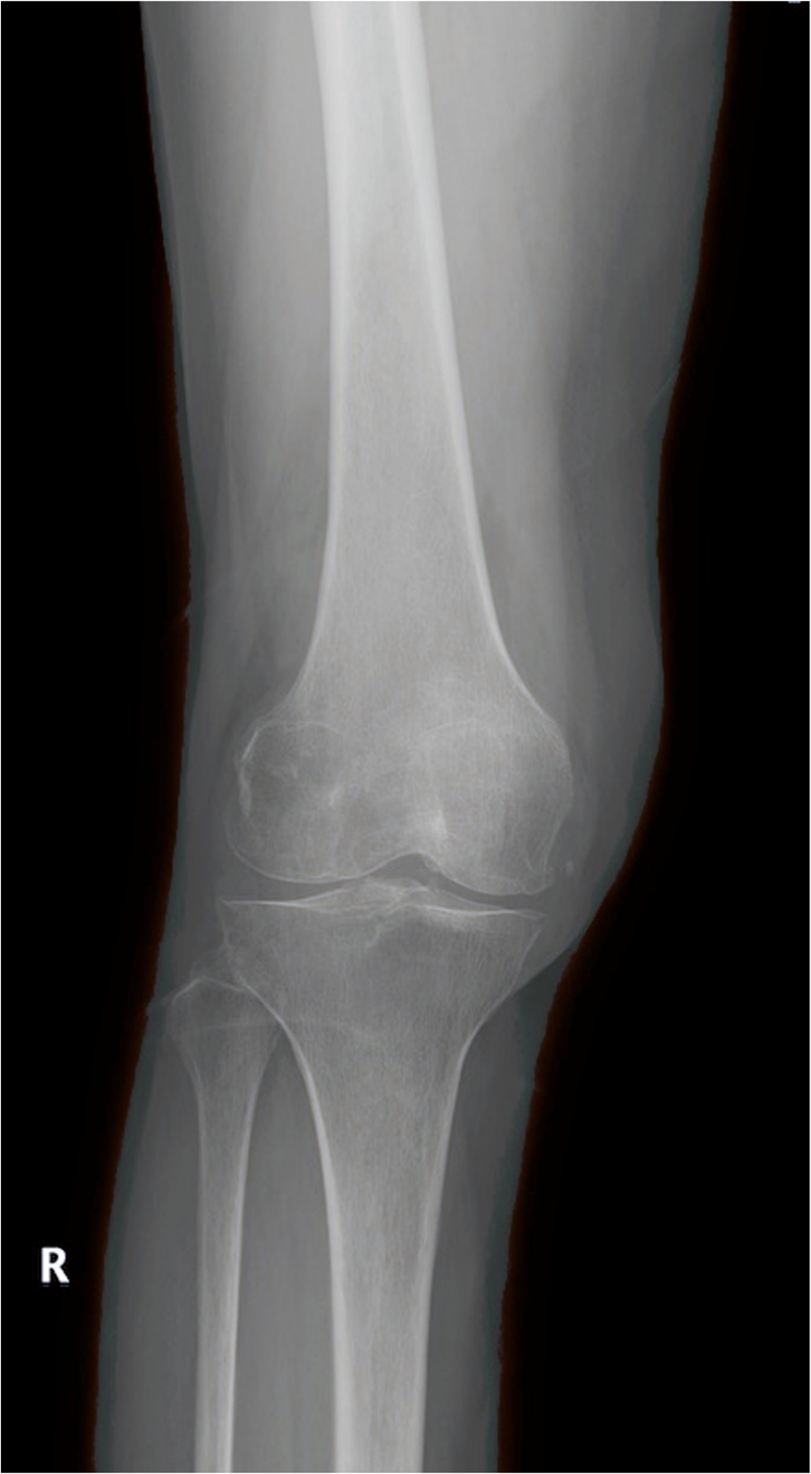
Typical X-ray findings of PVNS in the knee

All patients with PVNS had knee pain before receiving CR TKA. In our study, seven patients (41%) underwent at least one synovectomy, five patients (29.4%) were associated with patella dislocation or subluxation, and ten patients (59%) had limited knee mobility with an average range of motion (ROM) of 89° (range 78–101°). The X-ray showed typical features of the end-stage knee joint in patients with PVNS, including narrowing of joint space and cystic destruction (Fig. 1).

### Surgical treatment

All surgeries were performed by the same surgeon and accomplished through the standard midline skin incisions and medial parapatellar arthrotomy. For patients with PVNS, the pathological synovium was radically resected and histopathologically assessed. Firstly, the proximal tibia was resected on the coronal plane perpendicular to the tibial axis, and the sagittal plane was inclined backwards by 6–8°, preserving the PCL. The distal femur was excised with 5–7° valgus; for patients with poor patella track, we appropriately increased the external rotation osteotomy of the femur. We provide sufficient space between the femur and tibia for synovectomy. All patients received the same type of CR prosthesis (LINK, Germany, Gemini MK II). There were no restrictions placed on ROM, activity postoperatively, or weight-bearing status.

### Outcome measures

Assessments were performed by a senior orthopedic surgeon who did not attend the treatments. The demographics in regard to age, sex, BMI, follow-up time, disease recurrence, revision rate, and wound complications were examined.

The ROM, Knee Society Scores (KSS) [12], and the survivorship curve of Kaplan-Meier implant were compared between the two groups. All data were assessed before surgery and the last follow-up after surgery.

The standard anteroposterior and lateral radiographs were used for all preoperative and postoperative radiologic evaluations.

### Statistical analysis

The normality of the continuous variables was checked with the Shapiro-Wilk test. If the data were normally distributed, the variables were checked with Student’s *t* test; if not, a non-parametric test was selected. Categorical variables were checked with Fisher’s exact test or chi-square test. The implant survivorship was generated with 95% confidence intervals (CI) by the Kaplan-Meier method. The data were analyzed with the SPSS 19.0 (IBM, Chicago, IL, USA). A *p* < 0.05 was considered significant.

## Results

**Fig. 2:**
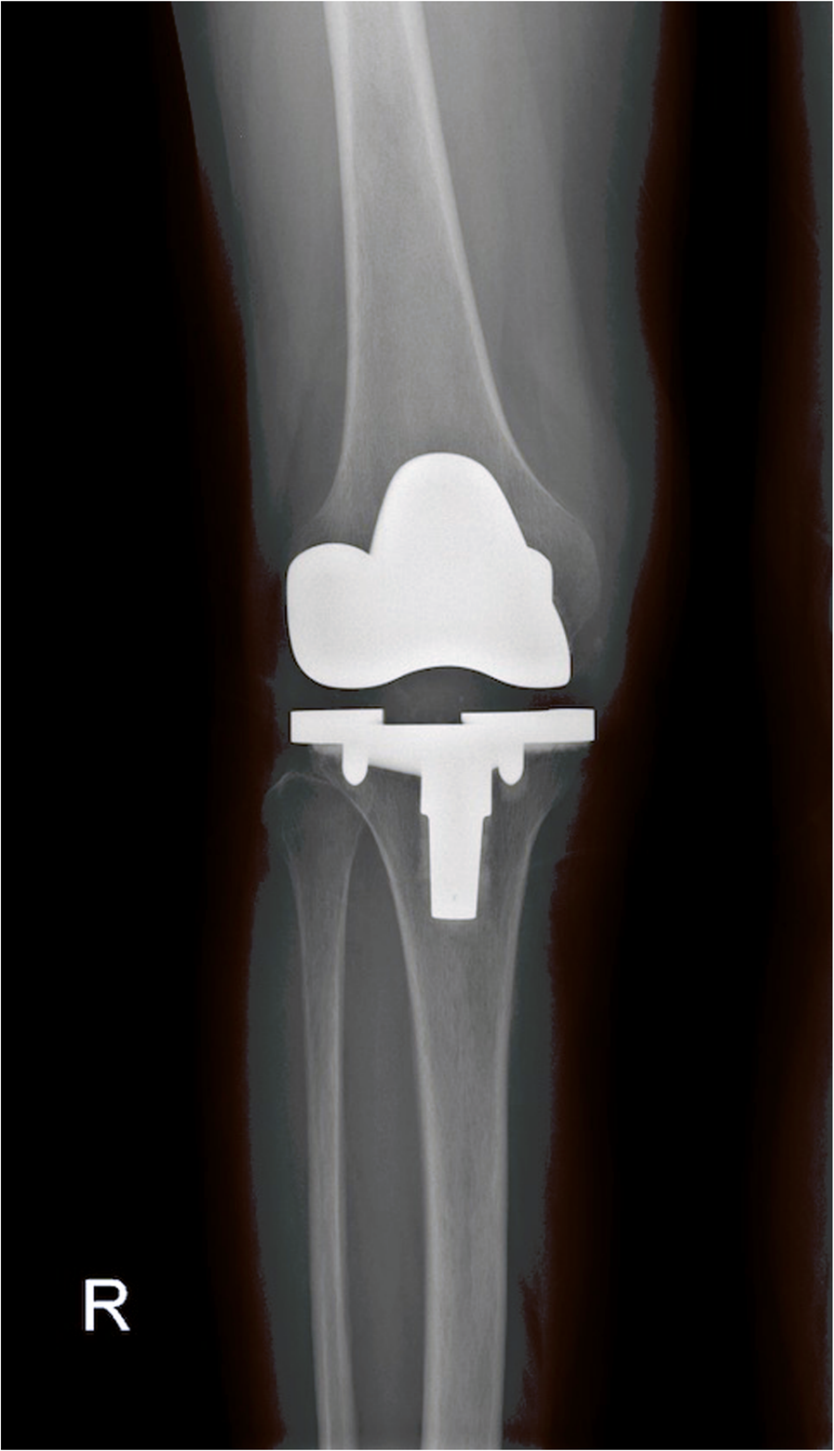
A radiograph of PVNS was taken 6.2 years after TKA showed well-fixed prosthesis

**Fig. 3:**
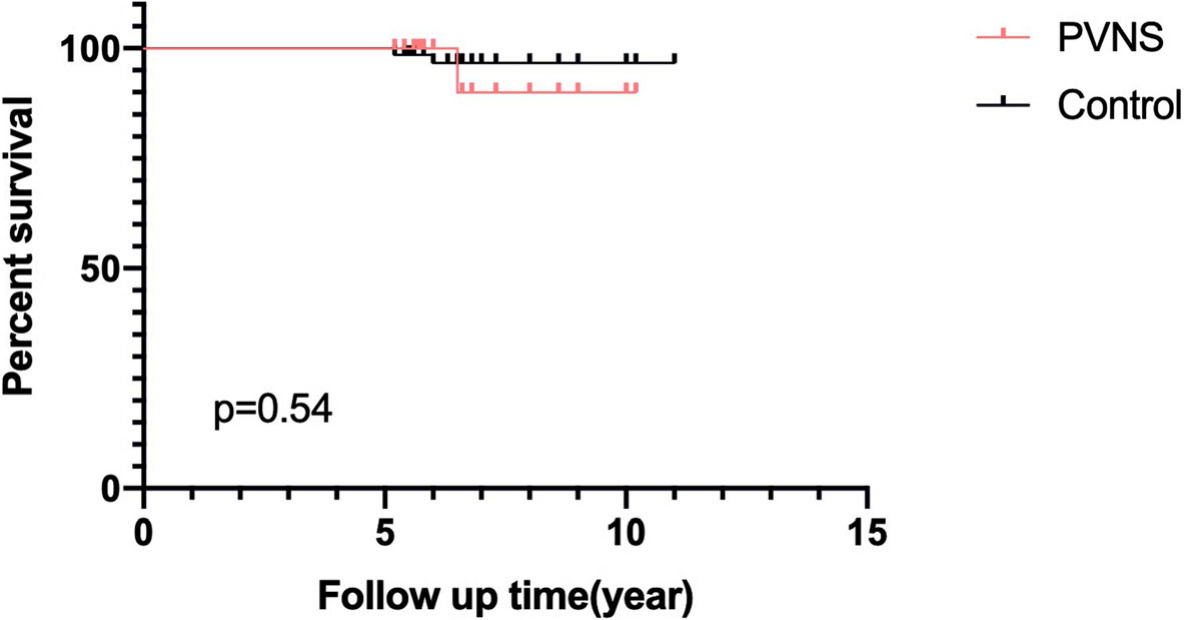
The Kaplan-Meier implant survivorship curve of patients in the two groups

All patients were followed up at least 5 years (Table 1). During the last follow-up, no clinical or radiological signs of prosthetic loosening were found in the PVNS group, indicating no recurrence of PVNS (Fig. 2). There was no significantly difference in ROM and Knee Society clinical and functional scores between the two groups before surgery and at the last follow-up after surgery (Table 2). All patients were able to exercise moderately without crutches. In the PVNS group, one patient underwent revision surgery because of periprosthetic fracture, and three patients had stiffness one year after surgery (17.6% vs 1.5%, *p* = 0.005; ROM:16–81°), but no revision was needed. In the control group, two patients underwent revision (one unstable revision and one infection) (*p* = 0.56). At 7 years, the implant survivorship without any revision was 90.0% in the PVNS group and 96.6% in the control group; however, there was no significant difference between the two groups (*p* = 0.54) (Fig. 3).

**Table 1:** Patient demographics in the two groups

	PVNS (*n* = 17)	Controls (*n* = 68)	*p* value
Age (years)	58.6 ± 7.2	59.2 ± 6.8	0.87
Sex, *n* (%)			0.80
Female	13 (76.5%)	50 (73.5%)	
Male	4 (23.5%)	18 (26.5%)	
BMI (kg/m^2^)	25.4 ± 3.3	25.7 ± 3.1	0.76
Follow-up (years)	7.2 ± 1.7	7.3 ± 1.8	0.86

Mean ± standard deviation

*PVNS* pigmented villonodular synovitis, *BMI* body mass index

**Table 2:** The outcomes following CR TKA in the two groups

	PVNS (*n* = 17)	Controls (*n* = 68)	*p* value
Pre-operative ROM	96 ± 5.2	97 ± 5.4	0.81
Last follow-up ROM	106 ± 9.7	107 ± 8.9	0.63
Pre-operative Knee Society clinical score	36 ± 3.2	37.9 ± 2.7	0.43
Last follow-up Knee Society clinical score	93.5 ± 3.8	93.6 ± 1.9	0.82
Pre-operative Knee Society functional score	37.9 ± 2.7	36.7 ± 3.3	0.50
Last follow-up Knee Society functional score	88.2 ± 1.4	88.1 ± 1.6	0.78
Recurrence, *n* (%)	0 (0%)	0 (0%)	–
Infection, *n* (%)	0 (0%)	1 (1.5%)	0.61
Chronic soft tissue pain, *n* (%)	1 (5.9%)	2 (2.9%)	0.56
Stiffness, *n* (%)	3 (17.6%)	1 (1.5%)	0.005
Abnormal patella track, *n* (%)	0 (0%)	1 (1.5%)	0.61
Any revision, *n* (%)	1 (5.9%)	2 (2.9%)	0.56

Mean ± standard deviation

*PVNS* pigmented villonodular synovitis, *ROM* range of motion

## Discussion

This study showed that the patients with PVNS who underwent CR TKA had similar survival rate and functional outcomes as the patients with OA who underwent CR TKA. In the minimum 5 years follow-up, no infection, osteolysis, and knee instability were found in the patients with PVNS. Furthermore, there was no evidence of the PVNS recurrence. However, these patients should pay more attention to the occurrence of postoperative stiffness complications.

The PVNS is a rare benign proliferative knee joint disease. Although the pathogenesis of PVNS remains unclear, some researchers believe that traumatic bleeding may be one of the causative factors [13, 14]. In a previous case report, the localized pigmented villonodular synovitis presents as recurrent dislocation of the patella [15]. This is consistent with our study; in our study, five patients (29.4%) with PVNS had a history of patella dislocation or subluxation. And, we appropriately increased the external rotation osteotomy of the femur in surgery for those patients. In the postoperative follow-up, these patients achieved good postoperative clinical outcomes and no abnormal patella track, and only one patient with anterior knee pain was found. Therefore, we believed that the patella dislocation or subluxation may be a susceptible factor for the knee PVNS. In the future, we should pay more attention to these patients.

The local knee PVNS is widely present in the anterior chamber of the knee, and the patients who have symptomatic are usually treated with arthroscopic debridement. However, the diffuse PVNS requires combined surgery, either through arthroscopy or open surgery [3, 16]. If the PVNS extends beyond the joint, an open surgery is required [16]. Although open surgery can reduce the local recurrence rate of diffuse PVNS, these procedures may lead to a high incidence of knee stiffness after surgery [17]. The ROM after TKA is associated with preoperative knee ROM [18], and in our study, we found that in the patients with PVNS who underwent open synovectomy, the knee ROM was poor after CR TKA. In addition, we also found that the incidence of postoperative knee stiffness in patients with PVNS was higher than the patients with OA, even if they have not received other surgeries before TKA, so we should pay more attention to the postoperative rehabilitation of patients with PVNS.

It is generally known that TKA is the most effective treatment for end-stage PVNS in patients who have severe OA due to PVNS progression, and the recurrence and revision rate of PVNS are lower than that with simple synovectomy [9]. The long-term results of TKA treatment with PVNS have been well recorded, and some studies have reported excellent long-term survival of TKA in patients with PVNS [9, 10]. However, it is still controversial to retain or substitute the PCL in TKA when treated with the patients with PVNS, and due to limited case studies of the knee PVNS, there is currently little data on the results of CR TKA in these patients.

During primary TKA, two principal designs are used: CR TKA and posterior-stabilized (PS) TKA. Compared with PS TKA, the CR TKA has been widely used because it improves the knee’s ability to exercise, preserves the knee’s proprioception, and increases the knee ROM and stability during knee extension and flexion [19, 20]. Although the PVNS and the rheumatoid arthritis have different types of inflammation and mechanisms of joint destruction, they all produce chronic inflammation environment in joints, so the two diseases have some comparability to some extent [21]. Scott and his colleagues [22] pointed out that 95% of rheumatoid arthritis patients had complete PCL during TKA surgery and believed that the PCL should be preserved during surgery to maximize femoral rollback. In addition, it has been informed that satisfactory clinical and radiological outcomes have been obtained in the rheumatoid arthritis patients who were followed up for an average of 10.5 years with CR TKA [23]. Miller [24] evaluated long-term outcomes of patients with rheumatoid arthritis who were followed up for 20 years after CR TKA; for any reason, the 20-year implant survival rate was 69%. They believe that the PCL dysfunction is rarely the cause of surgical failure [24]. This is consistent with our present study. In our study, the patients with PVNS who underwent CR TKA achieved excellent mid-term follow-up outcomes.

The PVNS most frequently affects the knee; although there were long-term follow-up studies and short-term complications in the previous literature with the evaluation of PVNS in arthroplasty, there was no clear middle-term follow-up control study. The previous TKA treatment in patients with PVNS was a minor cohort study, primarily to assess implant survival and function, and did not quantify the risk of postoperative complications, which may be due to the fewer patients [9, 25]. Although Houdek et al. [9] did not compare the incidence of complications to the control group, the most common complication in their study was the loss of knee ROM, which was similar to our study. According to the previous reports, the revision rate of the patients with PVNS was as high as 21%, which was significantly higher than the incidence of published primary TKA for OA [9, 26]. In our study, only one patient underwent revision because of periprosthetic fracture. In addition, the implant survivalship without any correction for 7 years after CR TKA was 90.0%, and we did not find any local recurrence. These clinical outcomes were similar to the patients with OA who underwent CR TKA. However, the revision rate may become inconsistent with additional long-term follow-up.

Radiotherapy and chemotherapy may be a viable option when surgery fails to eradicate PVNS or recurrence. Medium-dose external irradiation (30–35 Gy) combined with surgical resection can reduce the recurrence rate in patients with extensive or invasive diseases [27, 28]. In recent years, significant advances have been made in the treatment of diffuse PVNS [29, 30]. Since PVNS often overexpresses colony-stimulating factor 1 (csf1), receptor-targeted chemotherapeutic drugs (csf1r) may be an effective treatment [29, 30]. Although these drugs were not used in patients in this series of study, it was believed that the young patients should consider using them to alleviate symptoms and delay TKA for as long as possible.

Our study had several limitations. First, this was a small sample retrospective study, which had its potential bias and weaknesses. A prospective study should be established to objectify these findings. Second, because the patients with PVNS in our study were treated with CR TKA, we were unable to compare the efficacy of different prostheses, such as PS TKA, semi-constrained, or rotating hinge prostheses.

## Conclusions

This study demonstrated that the function of patients with PVNS who underwent CR TKA had been significantly improved, and the survival rate of implants in these patients was similar to the patients with OA. Consequently, the patients with PVNS who underwent CR TKA might be an achievable option. However, these patients should pay more attention to the occurrence of postoperative stiffness complications.

## Data Availability

The detailed data and materials of this study were available from the corresponding author through emails on reasonable request.

## Abbreviations

PVNS: Pigmented villonodular synovitis
OA: Osteoarthritis
TKA: Total knee arthroplasty
CR: Cruciate-retaining
PS: Posterior-stabilized
BMI: Body mass index
ROM: Range of motion
KSS: Knee Society Score
PCL: Posterior cruciate ligament
